# The Dorsomedial Hypothalamus Is Involved in the Mediation of Autonomic and Neuroendocrine Responses to Restraint Stress

**DOI:** 10.3389/fphar.2019.01547

**Published:** 2020-01-23

**Authors:** Taíz F. S. Brasil, Silvana Lopes-Azevedo, Ivaldo J. A. Belém-Filho, Eduardo A. T. Fortaleza, José Antunes-Rodrigues, Fernando M. A. Corrêa

**Affiliations:** ^1^ Department of Pharmacology of the School of Medicine of Ribeirão Preto, University of São Paulo, Ribeirão Preto, Brazil; ^2^ Department of Physiology of the School of Medicine of Ribeirão Preto, University of São Paulo, Ribeirão Preto, Brazil

**Keywords:** dorsomedial hypothalamus, restraint stress, cardiovascular response to stress, neuroendocrine response, CoCl_2_

## Abstract

We hypothesized that dorsomedial hypothalamus (DMH) modulates autonomic and neuroendocrine responses in rats at rest and when subjected to restraint stress (RS). Male Wistar rats were used, and guide cannulas were bilaterally implanted in the DMH for microinjection of vehicle or the nonspecific synaptic blocker CoCl_2_ (1 mM/100 nl). A polyethylene catheter was inserted into the femoral artery for the recording of arterial pressure and heart rate (HR). Tail temperature was measured using a thermal camera. The session of RS started 10 min after DMH treatment with vehicle or CoCl2. Under home-cage condition, the pretreatment of DMH with CoCl_2_ increased baseline blood pressure (BP), and heart rate (HR) without affecting the tail temperature. In addition, it decreased plasma vasopressin levels without affecting plasma corticosterone and oxytocin contents. When rats pretreated with CoCl_2_ were exposed to RS, the RS-evoked cardiovascular were similar to those observed in vehicle-treated animals; however, because cobalt pretreatment of the DMH increased baseline BP and HR values, and the RS-evoked cardiovascular responses did not exceed those observed in vehicle-treated animals, suggesting a possible celling limit, the possibility that DMH is involved in the modulation of RS-evoked cardiovascular responses cannot be certainly excluded. Nonetheless, the pretreatment of DMH with CoCl_2_ blocked the reduction in tail temperature caused by RS. The DMH pretreatment with CoCl_2_ did not modify the RS-evoked increase in plasma corticosterone and oxytocin contents. In conclusion, the present data suggest the involvement of DMH in the maintenance of BP, HR, and vasopressin release under the rest conditions at the home-cage. Furthermore, indicate that DMH is an important thermoregulatory center during exposure to RS, regulating tail artery vasoconstriction.

## Introduction

The dorsomedial hypothalamus (DMH) is a brain structure adjacent to the third ventricle, which is located caudal and ventral to the paraventricular nucleus of the hypothalamus (PVN). Its lateral portion is adjacent to both the fornix and the lateral hypothalamic area. The DMH is subdivided into two distinct portions: a diffuse and a compact zone (Paxinos and Watson, 2007).

The involvement of DMH in cardiovascular regulation has been well evidenced in the literature. Electrical stimulation or chemical inhibition of this area has been reported to increase blood pressure (BP) and heart rate (HR) in anesthetized rats (Soltis and DiMicco, 1991; Li et al., 2018). In addition, the DMH also participates in neuroendocrine regulation, because its chemical stimulation increases the release of adrenocorticotropic hormone (ACTH), as well as the expression of *c-fos* protein in the PVN, causing a response similar to that observed under stressful situations (Bailey and Dimicco, 2001; Morin et al., 2001; Rusyniak et al., 2008). The PVN is well recognized as an important structure for the integration of neuroendocrine and autonomic responses; PVN neuroendocrine parvocellular neurons modulate ACTH release, while PVN magnocellular neurons synthesize vasopressin and oxytocin (Lind et al., 1985). Emotional stress is well recognized as an integrated physiological response of the neuroendocrine and autonomic nervous systems, and has been related to the development of different pathologies in humans, such as hypertension, arrhythmias, myocardial infarction, as well as psychiatric disorders (Henry et al., 1986; Folkow, 1987; Meerson, 1994; Nalivaiko, 2011). The RS is defined in the literature as an animal stress model, which causes sustained BP and HR increase, reduction in tail temperature, activation of the hypothalamic-pituitary adrenal axis (HPA), and has been extensively used to study the autonomic and neuroendocrine responses to stress (Barron and Van Loon, 1989; Krieman et al., 1992; Irvine et al., 1997; Bhatnagar et al., 1998; McDougall et al., 2000; Brasil et al., 2018).

The DMH is an important center of regulation of the emotional response to stress (DiMicco et al., 2002). It was demonstrated that foot-shock stress activates neuronal projections from the DMH to PVN (Li and Sawchenko, 1998), and that local pharmacological manipulations alter the cardiovascular response triggered by stress (Stotz-Potter et al., 1996b), including the restraint stress model (RS) (Stamper et al., 2017). Although there is this study about the involvement of DMH on RS-related responses (Stamper et al., 2017), the role of the DMH in the modulation of different responses caused by acute RS it is not well established in the literature. In this context, we hypothesize that DMH modulates autonomic (BP, HR, and tail temperature) and neuroendocrine responses (corticosterone, vasopressin, and oxytocin release) both under rest conditions as well as during stressful situations.

For this purpose, we pretreated the DMH of unanesthetized rats with the nonspecific and acute synaptic blocker CoCl_2_ (Kretz, 1984; Lomber, 1999), and analyzed the effect of its inactivation on the autonomic and neuroendocrine function under rest and stressful conditions.

## Experimental Procedures

### Animals

All experimental procedures were carried out following protocols approved by the University of São Paulo Animal Ethical Committee (n° 221/2018), which complies with the European Communities Council Directive of September 22, 2010 (82,010/63/EU). Male Wistar rats weighing 250–280g were used. Animals were housed in plastic cages in a temperature-controlled room at 25°C in the Animal Care Unit of the Department of Pharmacology, School of Medicine of Ribeirão Preto, University of São Paulo, Brazil. Animals were kept under a 12–12 h light-dark cycle (lights on between 06:00 and 18:00 h). Animals had free access to water and standard laboratory food. The rats were transported to the experimental room and remained in their own cages until being subjected to restraint. Experiments were performed during the morning period (08:00 to 12:00) in order to minimize possible circadian rhythm interferences.

### Surgical Procedures

Seven days before the experiment, animals were anesthetized with 10% ketamine–2% xylazine (0.09 ml/100 g body weight, i.p.), and their heads were fixed to a stereotaxic apparatus (Stoelting, USA). After local anesthesia of the scalp with 2% lidocaine, the skull was surgically exposed and trepanned with a dental drill at 1.9 mm from the medial line and 7.2 mm anterior to the interaural line (Paxinos and Watson, 2007). Stainless steel guide cannulas (26 G) were implanted bilaterally into the DMH based on the rat brain atlas of Paxinos and Watson (2007): AP = -3.1 mm anterior to the interaural line, L = 2.3 mm from the medial suture, V = -7.2 mm from the skull at a 12° angle. The incisor bar was positioned at -3.2 mm. Guide cannulas were implanted 1 mm above the intended injection sites and fixed to the skull with a metal screw and dental cement. Stainless wires with 0.2 mm outer diameter and 15 mm in length were introduced into the cannulas to avoid occlusion during the recovery period after surgery. After the surgery, animals were treated with poly-antibiotic, with streptomycins and penicillins (Pentabiotico^®^, FontouraWyeth, Brazil, 80.000 UI i.m.) to prevent infection and the non-steroidal anti-inflammatory flunixin meglumine (Banamine^®^, Schering Plough, RJ, Brazil) for post-operation analgesia. One day before the experiment, a polyethylene catheter was inserted into the femoral artery under anesthesia, for mean arterial pressure (MAP) and HR recording. The catheter was exposed on the dorsum of the animals and fixed to the skin. Cardiovascular and temperature recordings were performed in their own cages or during the session of restraint. After surgical procedures the animals received flunixin meglumine (Banamine^®^, Schering Plough, Brazil; 2.5 mg/kg s.c.).

### Cardiovascular Recording

On the day of the experiment, the arterial catheters were connected to a pressor transducer. The pulsatile arterial pressure (PAP) was recorded using an amplifier (model 7754A, Hewlett Packard, USA) coupled to a computerized system (MP100A, Biopac, USA). MAP and HR values were derived from PAP data using the Acknowledge III software (Biopac, USA). The HR (beats/min, bpm) was calculated from PAP peak intervals integrated every 6 s.

### Tail Temperature Measurement

Tail temperature was measured throughout the entire rat’s tail using a thermal camera (Multi-Purpose Thermal Imager IRI 4010; Infrared Integrated Systems Ltd., USA), with the camera positioned at a distance 50 cm above the tail. Tail temperature value was averaged from ten points distributed throughout the tail’s length.

### Acute Restraint Stress

Animals were subjected to the session of restraint by placing each animal into a plastic cylindrical tube (6.5 cm diameter, 15 cm length), ventilated by holes (1 cm diameter) that comprised approximately 20% of the tube surface. Restraint session lasted 60 min. Animals were subjected to only one session of restraint, in order to avoid habituation.

### Neuroendocrine Protocol and Plasma Corticosterone, Vasopressin, and Oxytocin Measurements

The neuroendocrine experiments were performed using the same protocol of RS. Briefly, rats were subjected to stereotaxic surgery and recovered for five days. One day before the experiment, rats were placed in individual home-cages and transported to the experimental room. During the experimental period, rats remained in their own cages until being subjected to restraint. Rats received one microinjection of CoCl_2_ or vehicle bilaterally into the DMH, and 10 min after, were subjected to one session of 0, 20, or 40 min of RS, in order to evaluate the time-course of the neuroendocrine response. Immediately after the RS session, rats were decapitated and the blood samples were collected from the trunk in heparinized tubes (10µl/ml). The plasma samples were separated by centrifugation (3,000 rpm for 15 min at 4°C) and stocked at -20°C. Plasma corticosterone, vasopressin and oxytocin were measured by radioimmunoassay according to established techniques, previously described in detail (Ruginsk et al., 2013).

### Drugs and Solutions

CoCl_2_ (1 mM/100 nl) (Sigma, USA) was dissolved in artificial cerebrospinal fluid (ACSF), which had the following composition: 100 mM NaCl, 2 mM Na_3_PO_4_, 2.5 mM KCl, 1mM MgCl_2_, 27 mM NaHCO_3_, 2.5 mM CaCl_2_, pH 7.4.Urethane (Sigma, USA) was dissolved in saline (0,9% NaCl). Flunixin meglumine (Banamine^®^, Schering Plough, Brazil) and the poly-antibiotic preparation of streptomycins and penicillins (Pentabiotico^®^, Fontoura-Wyeth, Brazil) were used as provided.

### Drug Microinjection Into the DMH

Bilateral injections were performed in a volume of 100 nl using a 1-µl syringe (7001KH; Hamilton, USA) connected to a microinjection needle (gauge G 33- Small Parts, USA) through a piece of PE-10 polyethylene tubing. The microinjection needle was 1 mm longer than the guide cannula. Microinjections were performed within a 5 s period. After microinjection, the needle was left in the guide cannula for 1 min before being removed. Drugs were prepared before the experiments and stored at -20°C. On the day of the experiment, drugs were thawed and kept on ice during experimental procedures.

### Histological Determination of Microinjection Sites

At the end of experiments, animals were anesthetized with urethane (1.25 g/kg i.p.) and 100 nl of 1% Evans blue dye was injected into the brain as a marker of the injection site. They were then subjected to intracardiac perfusion with 0.9% NaCl followed by 10% formalin. Brains were removed and post-fixed for 48 h at 4°C, and serial 40 µm-thick sections were cut with a cryostat (CM1900, Leica, Germany). Sections were stained with 1% cresyl violet, for light microscopy analysis. The placement of the microinjection needles was determined by analyzing serial sections, and was identified according to the rat brain atlas of Paxinos and Watson (2007).

### Data Analysis

Statistical analysis was performed using the Prism software. Data were expressed as means ± SEM. The raw basal values of MAP and HR was represented by the total mean of the ten points before and after the pretreatment. The raw basal values of tail temperature (TT) was represented by the total mean of three points before and after the pretreatment. The raw means of the cardiovascular values were represented by the total mean of sixty points during RS. The raw mean of the TT was represented by the total mean of seven points during RS. The Student’s paired t test was used to compare the raw basal values of MAP, HR, and TT before and after treatments. The Student’s unpaired t test was used to compare the raw basal values and RS average values between ACSF and CoCl_2_-treated animals. The changes from baseline within the groups were analyzed using the one-way ANOVA followed by Dunnet´s *post-hoc* test comparing each point to the first minute of the time-course (time -20 min). The time-course of changes in ΔMAP, ΔHR, and ΔTT was analyzed using repeated measures two-way ANOVA with treatment (vehicle and drug) as a main factor, and time as a repeated measurement. Treatment effects on RS-related responses (mean ΔMAP, ΔHR, and ΔTT) were further compared to the vehicle control using the Fisher’s *post-hoc* test. The neuroendocrine response was analyzed using the total mean of four time-course points, (-10, 0, 20, and 40 min) using two-way ANOVA followed by Fisher’s *post-hoc* test, with treatment (vehicle or CoCl_2_) and condition (home-cage or RS) as main factors. The unpaired t Student´s test with Welch´s correction was used to compare the treated group with the control group on the same conditions (home-cage or RS). Values of p < 0.05 were assumed as statistically significant differences between the groups. The effect sizes was calculated for the main effects using Stata 14 software and the data were expressed as Cohen’s *d* value for t tests and as omega squared (Ω^2^) for ANOVA.

## Results

**Figure 1:**
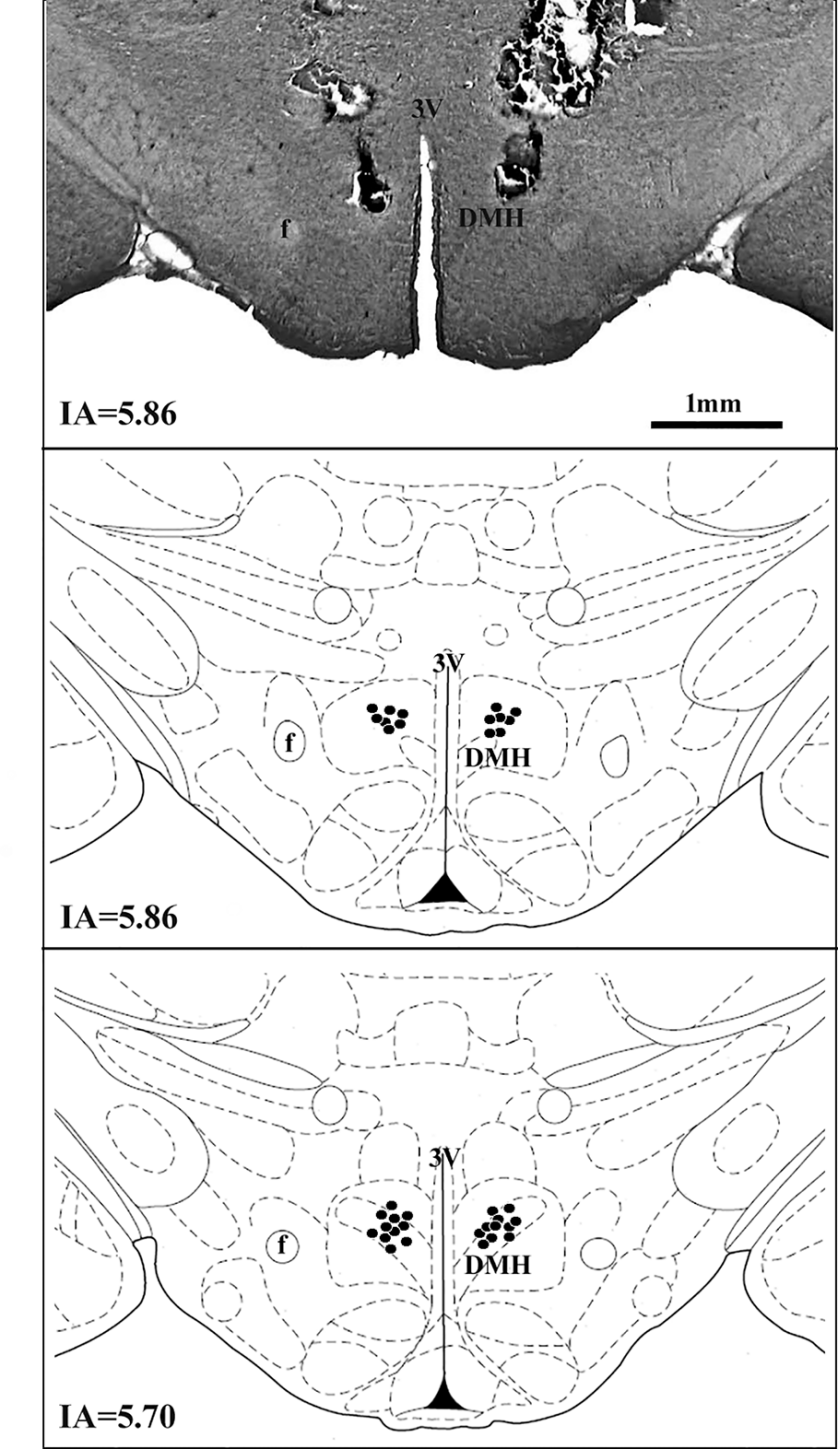
A diagrammatic representation based on the rat brain atlas of Paxinos and Watson (2007) indicating DMH microinjections sites (filled circles). The number of points represented can be fewer than the real number of animals used in the experiment due to overlap. DMH, dorsomedial hypothalamus; f, fornix; 3V, third ventricle; IA, interaural.

A diagrammatic representation showing the bilateral sites of microinjection in the DMH of all animals used is presented in **Figure 1**.

### Effect of Cocl_2_ Microinjection Into the DMH on the Autonomic Responses in Rats At the Home-Cage.

The analysis of raw basal values mean of ten points before and those after the pretreatment (-20 to -11 min *versus* -10 to -1 min) using the Student’s paired t test indicated that the microinjection of ACSF (100 nl) (n = 4) into the DMH did not affect the MAP (t = 1.5, P = 0.2), HR (t = 1.6, P = 0.2) and TT (t = 0.5, P = 0.6), when compared the basal values before and after the ACSF-treatment. The microinjection of CoCl_2_ (1 nM/100 nl) (n = 4) into the DMH significantly increased the MAP (t = 4.6, P = 0.01, Cohen’s *d* = -1.00) and HR (t = 4.3, P = 0.02, Cohen’s *d* = -1.56), when compared the basal values before and after the CoCl_2_-treatment, without affecting the TT one (t = 0.4, P = 0.6) (**Table 1**).

**Table 1:** Raw basal values of mean arterial pressure (MAP), heart rate (HR) and tail temperature (TT) before and after the microinjection of CoCl_2_ into the DMH in rats under the home-cage situation.

CoCl_2_ (mM/100nl)	n	MAP				HR				TT			
		Before ± SEM (mmHg)	After ± SEM (mmHg)	**t=**	**P=**	Before ± SEM (bpm)	After ± SEM (bpm)	**t=**	**P=**	Before ± SEM (°C)	After ± SEM(°C)	**t=**	**P=**
**0**	4	106.7 ± 1.6	108.6 ± 1.3	1.5	0.2	364.8 ± 12.4	375.6 ± 13.7	1.6	0.2	26.7 ± 0.7	27.5 ± 1.0	0.5	0.6
**1**	4	99.9 ± 3.9	108.8 ± 4.7	4.6	0.01*	373.6 ± 22.3	478.0 ± 41.4	4.3	0.02*	26.2 ± 0.8	25.9 ± 0.7	0.4	0.6

Values are raw mean and ± SEM. *significant difference before and after treatment, Student’s paired t test, P < 0.05. No significant difference was found in the MAP basal values of CoCl_2_-treated animals when compared to the ACSF one (0 versus 1) before (t = 1.5, P = 0.1) and after (t = 0.04, P = 0.9) pretreatment into DMH. No significant difference was found in the HR basal values of CoCl_2_-treated animals when compared to the ACSF one (0 versus 1) before (t = 0.3, P = 0.7) and after (t = 2.3, P = 0.08) pretreatment into DMH. No significant difference was found in the TT basal values of CoCl_2_- treated animals when compared to the ACSF one (0 versus 1) before (t = 0.5, P = 0.6) and after (t = 1.3, P = 0.2) pretreatment into DMH. Student’s unpaired t test), P > 0.05.

**Figure 2:**
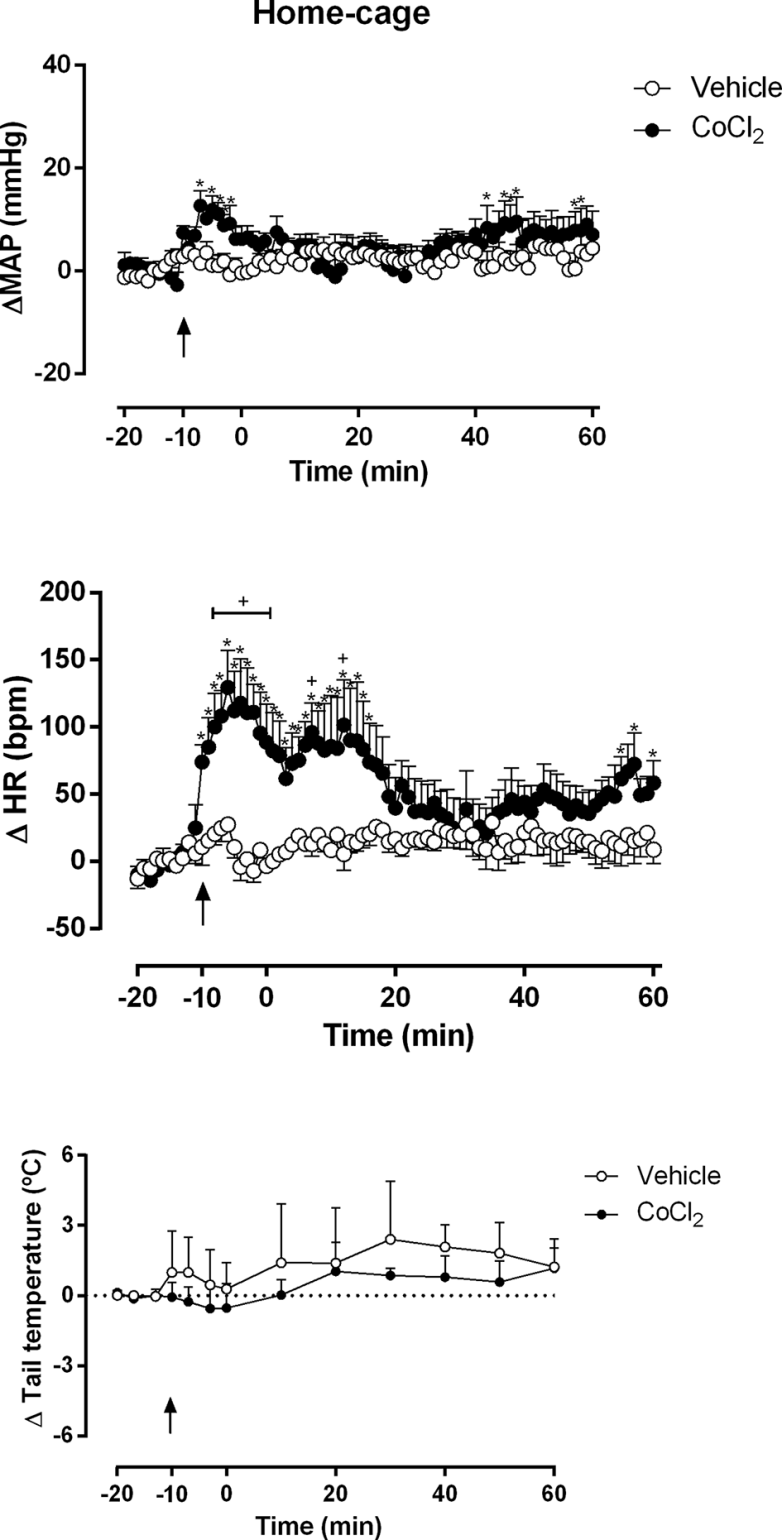
Mean arterial pressure (ΔMAP), heart rate (ΔHR) and tail temperature (Δ tail temperature) changes over time in rats during rest at home-cage with the DMH pretreated with either vehicle (ACSF) (open circles, n = 4) or CoCl_2_ (1 mM/100 nl) (filled circles, n = 4). Rats were treated at time -10 min. Points represent the mean and bars the SEM; arrow indicates the treatment; *significant difference between time-points from CoCl_2_-treated animals and vehicle-treated (ACSF), (two-way ANOVA followed by Fisher’s *post-hoc* test, P < 0.05). ^+^significant difference between the indicated point and the basal value at time -20 min in CoCl_2_-treated group (one-way ANOVA followed by Dunnet’s *post-hoc* test, P < 0.05).

The one-way ANOVA indicated that the microinjection of ACSF (100 nl) (n = 4) into the DMH did not affected the ΔMAP (F_80,__243_ = 0.60, P = 0.99, ω^2^ = 0) and ΔHR (F_80,__243_ = 0.75, P = 0.92) time-course within the group, when comparing each time point to the first minute (time -20 min). The one-way ANOVA indicated that the microinjection of CoCl_2_ (1 mM/100 nl) (n = 4) into the DMH increased the ΔMAP (F _80,__243_ = 1.41, P = 0.02, ω^2^ = 0.09) and ΔHR (F_80,__243_ = 2.15, P < 0.0001, ω^2^ = 0.22) time-course within the group, when comparing each time point to the first minute (time -20 min). The Dunnet’s *post-hoc* point-to-point test is presented in the figure (**Figure 2**).

The two-way ANOVA indicated that the microinjection of CoCl_2_ (1 mM/100 nl) (n = 4) into the DMH significantly increased the ΔMAP (treatment: F _(1,__486)_ = 48.87, P < 0.0001, ω^2^ = 0.086; time: F _(80,__486)_ = 1.196, P = 0.13,; interaction: F _(80,__486)_ = 1.03, P = 0.40) and ΔHR (treatment: F _(1,__486)_ = 228.7, P < 0.0001, ω^2^ = 0.318; time: F _(80,__486)_ = 2.080, P < 0.0001, ω^2^ = 0.132; interaction: F _(80,__486)_ = 1.757, P = 0.0002, ω^2^ = 0.096) parameters, when compared with ACSF-treated animals (n = 4) (**Figures 2A, B**). A representative figure of the cardiovascular changes caused by CoCl_2_ microinjection under the home-cage situation is shown in **Figure 4A**. Under rest condition at home-cage, the microinjection of CoCl_2_ (1 mM/100 nl) did not affect the ΔTT (treatment: F _(1,__78)_ = 2.59, P = 0.11; time: F _(12,78)_ = 0.56, P = 0.86; interaction: F _(12,__78)_ = 0.12, P = 0.99) when compared with the ACSF-treated animals The Fisher’s *post-hoc* point-to-point test is presented in the figure. (**Figure 2C**).

### Effect of Cocl_2_ Microinjection Into the DMH on the Cardiovascular Response in Rats Subjected to RS

The analysis of raw basal values mean of the ten points before and those after the pretreatment (-20 to -11 min *versus* -10 to -1 min) by the Student’s paired t test indicated that the microinjection of ACSF (100 nl) (n = 5) into the DMH did not affected the MAP (t = 1.9, P = 0.1), HR (t = 1.2, P = 0.2) and TT (t = 1.5, P = 0.1), when compared the basal values before and after the ACSF-treatment. The microinjection of CoCl_2_ (1 mM/100 nl) (n = 5) into the DMH significantly increased the MAP (t = 3.3, P = 0.02, Cohen’s *d* = -0.66) and HR (t = 3.7, P = 0.01, Cohen’s *d* = -1.85), when compared the basal values before and after the CoCl_2_-treatment, without affecting the TT one (t = 0.4, P = 0.6) (**Table 2**).

**Table 2:** Raw basal values of mean arterial pressure (MAP), heart rate (HR) and tail temperature (TT) before and after the microinjection of CoCl_2_ into the DMH in rats subjected to RS.

Baseline values before RS													
CoCl_2_ (mM/100nl)	n	MAP				HR				TT			
		Before ± SEM (mmHg)	After ± SEM (mmHg)	t=	P=	Before ± SEM (bpm)	After ± SEM (bpm)	t=	P=	Before ± SEM (°C)	After ± SEM(°C)	t=	P=
**0**	5	103.0 ± 2.9	101.6 ± 2.7	1.9	0.1	373.3 ± 14.2	384.3 ± 6.7	1.2	0.2	30.6 ± 0.7	29.6 ± 0.7	1.5	0.1
**1**	5	93.1 ± 4.9	100.9 ± 5.4	3.3	0.02**#**	349.3 ± 8.0	415.0 ± 21.1	3.7	0.01**#**	29.9 ± 1.1	30.2 ± 1.6	0.4	0.6
**Autonomic response during RS**													
**CoCl_2_ (mM/100nl)**	n	**MAP**				**HR**				**TT**			
		**RS average ± SEM (mmHg)**		**t=**	**P<**	**RS average ± SEM (bpm)**		**t=**	**P<**	**RS average ± SEM (°C)**		**t=**	**P<**
**0**	5	110.5 ± 0.5		16.9	0.0001*	442.7 ± 2.6		6.7	0.0001*	27.5 ± 0.2		7.5	0.0001*
**1**	5	100.3 ± 0.3				421.7 ± 1.6				30.1 ± 0.1	

Values are raw mean and ± SEM. **#**significant difference before and after treatment, Student’s paired t test, P < 0.05. No significant difference was found in the MAP basal values of CoCl_2_-treated animals when compared to the ACSF one (0 versus 1) before (t = 1.7, P = 0.1) and after (t = 0.10, P = 0.9) pretreatment into DMH. No significant difference was found in the HR basal values of CoCl_2_-treated animals when compared to the ACSF one (0 versus 1) before (t = 1.4, P = 0.1) and after (t = 1.4, P = 0.2) pretreatment into DMH. No significant difference was found in the TT basal values of CoCl_2_- treated animals when compared to the ACSF one (0 versus 1) before (t = 0.5, P = 0.6) and after (t = 0.3, P = 0.7) pretreatment into DMH. Student’s unpaired t test), P > 0.05. *significant difference when compared ACSF-RS and CoCl_2_-RS groups, Student’s unpaired t test, P < 0.05.

The Student’s unpaired t test indicated that the microinjection of CoCl_2_ reduced the pressor (t = 16.9, P < 0.0001, Cohen’s *d* = 24.7) and tachycardic (t = 6.7, P < 0.0001, Cohen’s *d* = 9.7) responses evoked by RS and impaired the reduction in TT (t = 7.5, P < 0.0001, Cohen’s *d* = -16.4), when compared to the ACSF-stressed group (**Table 2**).

**Figure 3:**
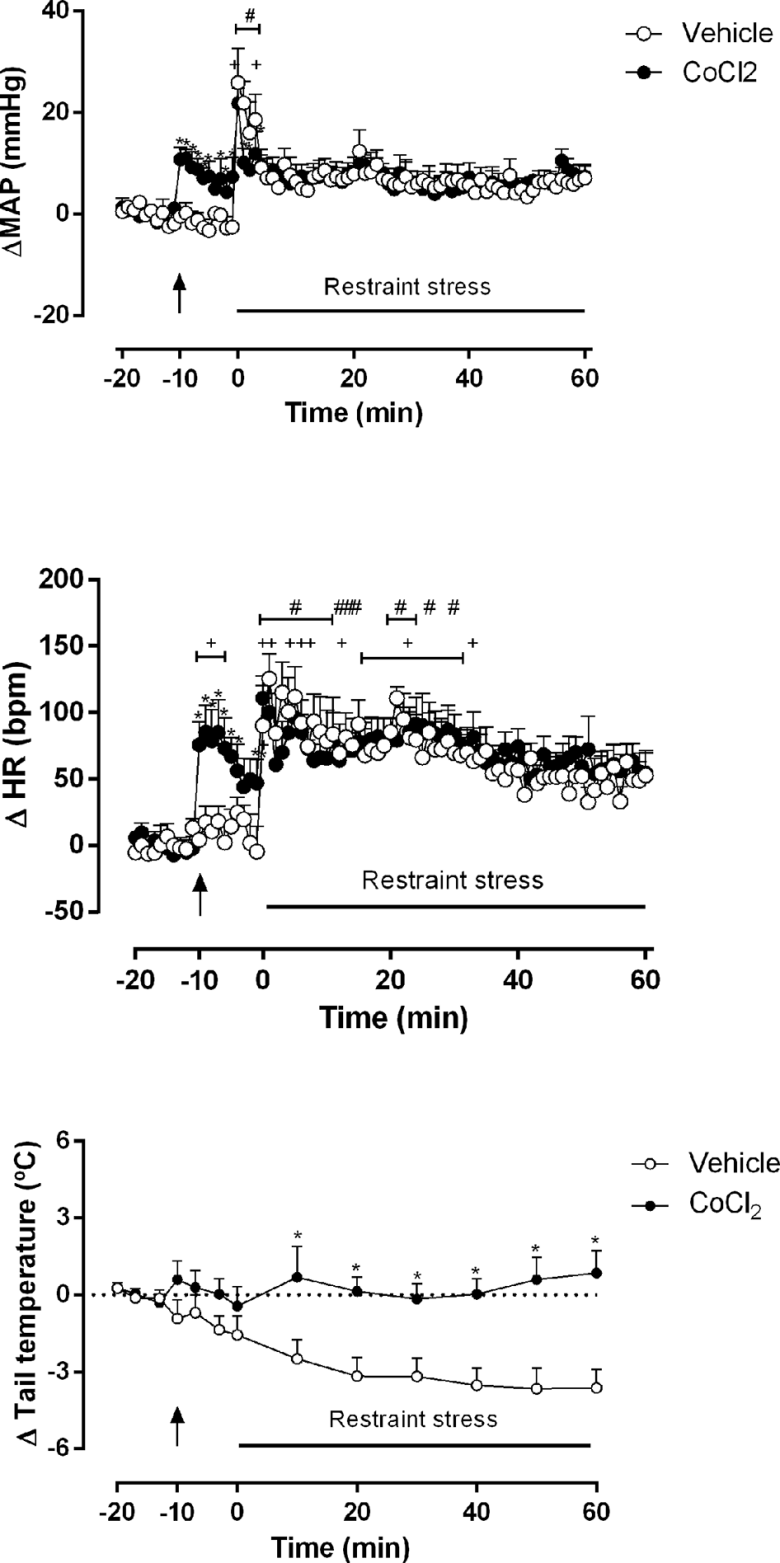
Mean arterial pressure (ΔMAP), heart rate (ΔHR) and tail temperature (Δ tail temperature) changes over time during acute restraint stress in rats with DMH pretreated with vehicle (ACSF) (open circles, n = 5) or CoCl_2_ (1 mM/100 nl) (filled circles, n = 5). Rats were subjected to acute restraint stress session at time 0 min. Points represent the mean and bars the SEM; arrow indicates the treatment; *significant difference between CoCl_2_-treated animals and the vehicle (ACSF) group, (two-way ANOVA followed by Fisher’s *post-hoc* test, P < 0.05). ^#^significant difference between the indicated point and the basal value at time -20 min in ACSF-treated group, ^+^significant difference between the indicated point and the basal value at time -20 min in CoCl_2_-treated group (one-way ANOVA followed by Dunnet’s *post-hoc* test, P < 0.05).

The one-way ANOVA indicated that the ACSF-RS group (100 nl) (n = 5) increased ΔMAP (F_80,__324_ = 3.48, P < 0.0001, ω^2^ = 0.33) and ΔHR (F_80,__324_ = 2.52, P < 0.0001, ω^2^ = 0.23) time-course within the group, when comparing each time point to the first minute (time -20 min). The one-way ANOVA indicated that the CoCl_2_-RS group (1 mM/100 nl) increased the ΔMAP (F_80,__324_ = 3.91, P < 0.0001, ω^2^ = 0.36) and ΔHR (F_80,__324_ = 3.30, P < 0.0001, ω^2^ = 0.31) time-course within the group, when comparing each time point to the first minute (time -20 min). The Dunnet’s *post-hoc* point-to-point test is presented in the figure (**Figure 3**).

**Figure 4:**
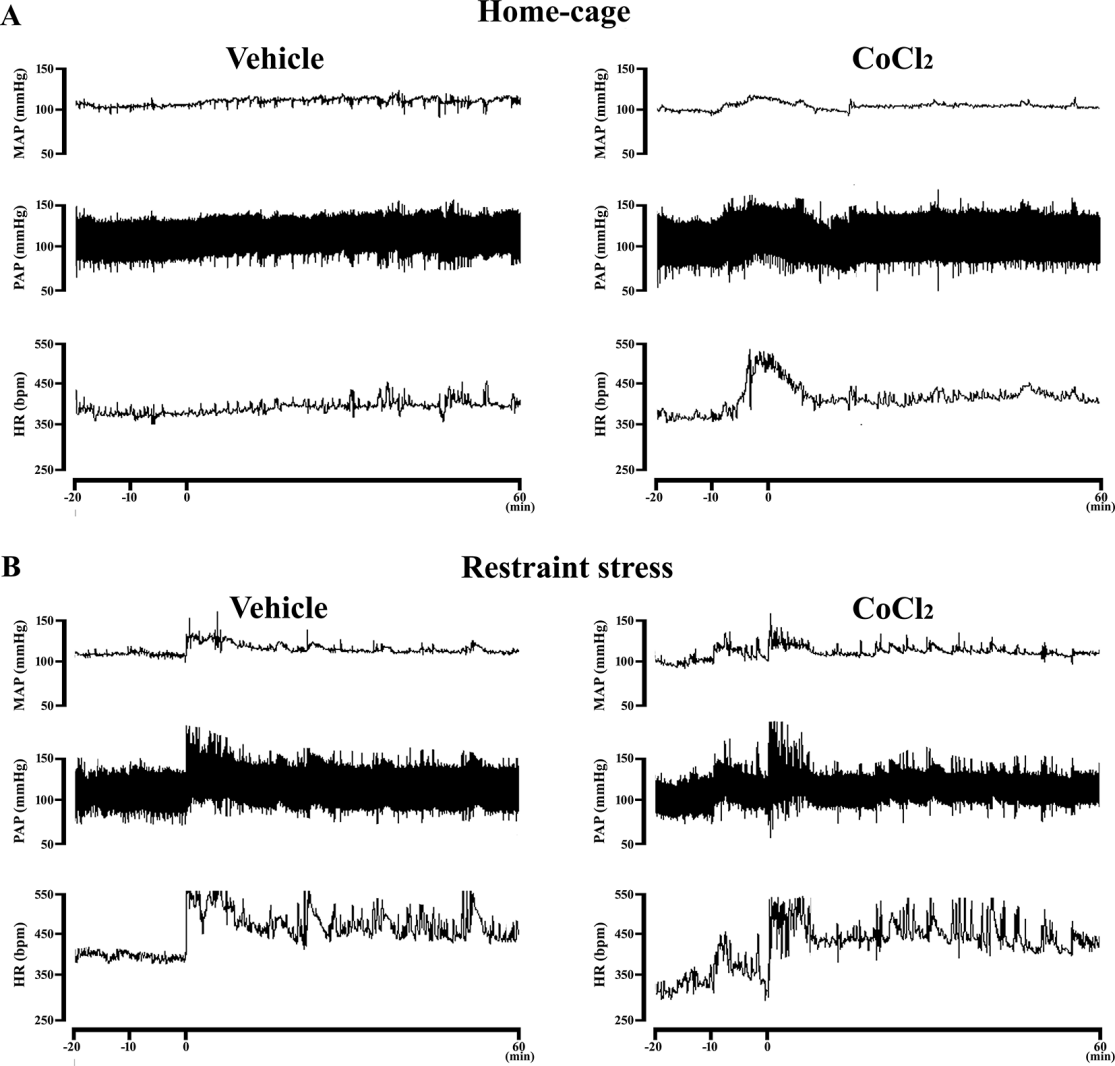
**(A)** Representative recording of one CoCl_2_-treated and one vehicle-treated (ACSF) animal demonstrating the pressor and tachycardic response caused by the microinjection of CoCl_2_ (1 mM/100nl) into the DMH of rats at rest in their home-cages. The microinjection was performed at time -10 min. **(B)** Representative recording of one CoCl_2_-treated and one vehicle-treated (ACSF) animals subjected to one RS session illustrating the cardiovascular changes caused by this stress model. The RS session was started at time 0 min and finished at 60 min.

The two-way ANOVA indicated that the microinjection of CoCl_2_ (1 mM/100 nl) (n = 5) into the DMH reduced the pressor (ΔMAP) (treatment: F(_1,__648)_ = 5.839, P = 0.01, ω^2^ = 0.007; time: F_(80,__648)_ = 4.914, P < 0.0001, ω^2^ = 0.30); interaction: F_(80,__648)_ = 1.13, P = 0.04, ω^2^ = 0.01) and the tachycardic (ΔHR) (treatment: F (_1,__648_) = 13.54 P = 0.0003, ω^2^ = 0.018; time: F _(80,__648)_ = 6.239, P < 0.0001, ω^2^ = 0.365); interaction: F _(80,__648)_ = 1,057, P = 0.35) responses during the RS session, when compared to the ACSF-stressed group (n = 5). A representative figure of the cardiovascular changes caused by RS is shown in **Figure 4B**. In addition, DMH pretreatment with CoCl_2_ (1 mM/100 nl) significantly impaired the reduction of ΔTT (treatment: F_(1,__104)_ = 63.49, P < 0.0001, ω^2^ = 0.373; time: F_(12,104)_ = 2.26, P = 0.013, ω^2^ = 0.115; interaction: F_(12,__104)_ = 3.08, P = 0.0009, ω^2^ = 0.177) caused by RS, when compared to the ACSF-stressed group. The Fisher’s *post-hoc* point-to-point test is presented in the figure. (**Figure 3**).

### Effect of Cocl_2_ Microinjection Into the DMH on the Neuroendocrine Response in Rats At the Home-Cage and Subjected to RS

**Figure 5:**
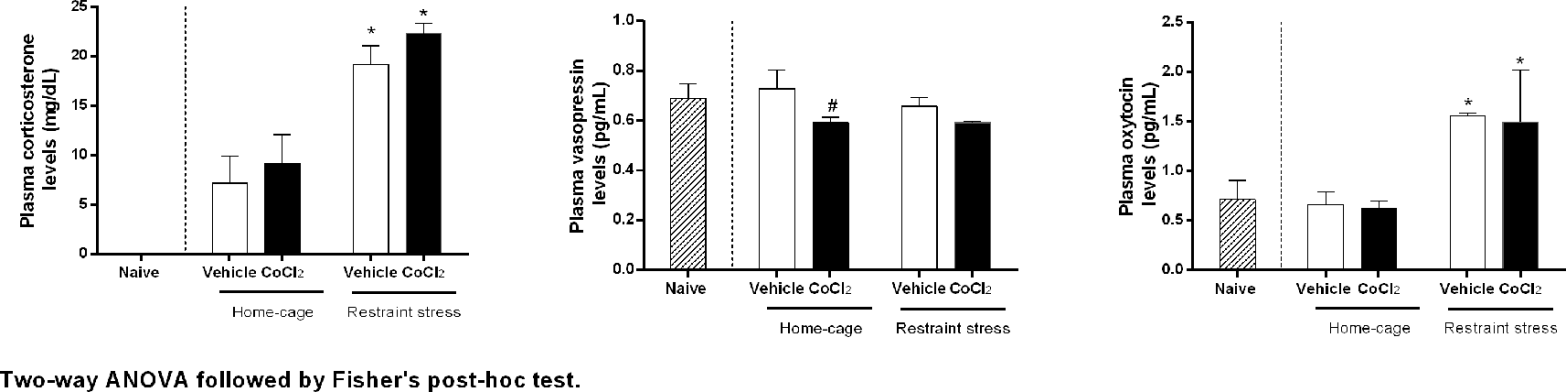
Corticosterone, vasopressin and oxytocin plasma concentrations over time before and during one RS session in rats with bilateral DMH pretreated with either vehicle (ACSF 100 nl n = 6) or CoCl_2_ (1 mM/100 nl n = 6). The RS session started at time 0 min and finished at 60 min. The dashed bars represent the naïve rats, white bars represents the DMH vehicle-treated (ACSF) rats, and the black bars represent DMH CoCl_2_-treated rats. Points represent the mean and bars the SEM. *indicates significantly difference between stressed-group and home-cage group, (Two-way ANOVA, p < 0.05). # indicates significantly difference between CoCl_2_-treated and vehicle-treated animals, both at home-cage condition (unpaired *t* Student test, P < 0.05).

The two-way ANOVA indicated that RS, regardless of treatment, significantly increased corticosterone (condition: F_(1,__20)_ = 63.45, P < 0.0001, ω^2^ = 0.75, interaction: F_(1,20)_ = 0.42, P = 0.52, ω^2^ = 0) and oxytocin (condition: F_(1,21)_ = 33.42, P < 0.0001, ω^2^ = 0.59 interaction: F_(1,21)_ = 0.23, P = 0.63) plasma levels, without affecting the vasopressin one (condition: F_(1,20)_ = 0.65, P = 0.42, interaction: _(1,20)_ = 1.05, P = 0.31). In addition, the pretreatment with CoCl_2_ into the DMH indicated that DMH pretreatment with CoCl_2_ (1 mM/100 nl) (n = 6) in animals at rest in home-cage, significantly reduced the basal plasmatic levels of vasopressin (treatment: F_(1,__20)_ = 5.05, P = 0.03, ω^2^ = 0.16), without affecting corticosterone (treatment: F_(1,__20)_ = 1.65, P = 0.21) and oxytocin (treatment: F_(1,__21)_ = 0.56, P = 0.46) levels, when compared to the ACSF home-cage group (n = 6) (**Figure 5**).

## Discussion

The DMH is recognized as an important area involved in central cardiovascular control. In the present study, we investigated the role of DMH in the control of the autonomic and neuroendocrine responses during two situations: at rest in the home-cage, and during exposure to RS, a well-established animal model of stress.

First, to study the participation of DMH in the maintenance of cardiovascular tonus and neuroendocrine parameters, we microinjected CoCl_2_, an acute and reversible synapses blocker, into the DMH of rats at rest in their home-cages. CoCl_2_ is a pharmacological approach to blockade the nonspecific synaptic transmissions, by reversible blockade of calcium channels of the presynaptic membrane and consequent prevention of the neurotransmitters release (Kretz, 1984). The microinjection of CoCl_2_ is an advantageous pharmacological tool to study the overall influence of a central area when compared to other inhibitors. The use of lidocaine, a local anesthetic, could block the local synapse transmission, but also the propagation of the action potencial by fibers of passage (Sandkühler et al., 1987). The lesion of a brain area is permanent, irreversible and also affect the fibers of passage (Lomber, 1999). The use of GABA_A_ antagonists could inhibit only the neurons that express GABA_A_ receptors (Martin and Ghez, 1999).

A previous study demonstrated that microinjection of 1 mM CoCl_2_ into the medial prefrontal cortex was able to interfere with a functional response, the barorreflex, in a similar way to the microinjection of lidocaine in the same area (Resstel et al., 2004), suggesting that this dose of CoCl_2_ is effective to blockade synaptic transmissions. Posteriorly, 1 mM CoCl_2_ was has been extensively used to study the involvement of brain areas in the neural circuitry of cardiovascular responses (Crestani et al., 2006; Tavares and Corrêa, 2006; Pelosi et al., 2007; Fortaleza et al., 2015; Fassini et al., 2017; Lopes-Azevedo et al., 2019).

The blockade of DMH synaptic transmissions by the pretreatment with CoCl_2_ caused an increase in baseline BP, HR, without affecting the tail temperature, suggesting an inhibitory influence of the DMH on the cardiovascular tonus. Previous studies have demonstrated that microinjection of bicuculine, a GABA_A_ antagonist, into the DMH caused a robust increase in HR and a less prominent increase in BP (Soltis and DiMicco, 1991; Bailey and Dimicco, 2001; Samuels et al., 2002). A possible explanation for the similarity of the results observed with the CoCl_2_ or bicuculine (Soltis and DiMicco, 1991; Bailey and Dimicco, 2001; Samuels et al., 2002) microinjection could be that DMH overall output negatively modulates areas involved in the neurocircuitry of the cardiovascular control, as evidenced by the overall inhibition of the local neurotransmission by the pretreatment with CoCl_2_. In this way, the increase in BP and specially in the HR, which is observed after the microinjection of the GABA_A_ antagonist bicuculine, could suggest the existence of a facilitatory mechanism in the DMH that is negatively modulated by neurons expressing GABA_A_ in that nuclei. As discussed by Lomber, 1999 there are several differences in the mechanisms of the inhibition approach used to study the role of a central area. Although inhibition with CoCl_2_ would indicated the overall resulting vector from multiple local neurotransmissions, the actual influence exerted by a particular neurotransmission would only be evidenced with the used of selective antagonists, such as the case of bicuculine, when concerning the gabaergic neurotransmission.

In order to evaluate the possible DMH participation in neuroendocrine modulation under a rest condition in the home-cage, we microinjected CoCl_2_ bilaterally into the DMH and measured plasma levels of corticosterone, vasopressin and oxytocin. Interestingly, the microinjection of CoCl_2_ into the DMH decreased basal vasopressin plasmatic levels, but did not affect basal corticosterone and oxytocin, suggesting a facilitatory role of DMH on the vasopressin release under rest condition. Previous studies have reported a DMH participation in different neuroendocrine responses. The microinjection of muscimol, a GABA_A_ agonist, into the DMH increased the ACTH plasma levels (Hunt et al., 2010), whereas DMH surgical lesion has been shown to decrease magnocellular vasopressin mRNA levels in the PVN (Kiss and Jezova, 2001), a result that corroborates the present findings with the inhibition of the DMH with CoCl_2_. This discrepancy of neuroendocrine results would take into account the different type of transmission inactivation caused by muscimol and by cobalt chloride or a DMH lesion. While cobalt and surgical lesions would silence overall DMH synaptic activity by either a presynaptic Ca^2+^ antagonism (Kretz, 1984; Lomber, 1999) or a physical lesion (Kiss and Jezova, 2001), the GABA_A_ agonist muscimol would mimic an increased gabaergic inhibitory influence on selective DMH neurons displaying GABA_A_ receptors (Martin, 1991); therefore, indicating that further studies are necessary to understand the influence played by individual neurotransmissions in the DMH on the cardiovascular modulation.

In a second set of experiments, we studied the possible involvement of DMH in the modulation of autonomic and neuroendocrine responses caused by acute exposure to RS. Animals were pretreated with CoCl_2_ or vehicle microinjected bilaterally into the DMH and then were subjected to RS, and results were compared with those observed in vehicle-treated animals. DMH inactivation by CoCl_2_ reduced the pressor and tachycardic response evoked by RS. Previous studies have reported that gabaergic inhibition of DMH, by local administration of muscimol, reduced the pressor and tachycardic response evoked by air jet stress (Soltis and DiMicco, 1992; DiMicco et al., 1996; Stotz-Potter et al., 1996a; de Menezes et al., 2008; Abreu et al., 2014). If the cardiovascular correlates of air jet and RS shares the same autonomic circuitry, the results from the use of muscimol (Soltis and DiMicco, 1992; DiMicco et al., 1996; Stotz-Potter et al., 1996a; de Menezes et al., 2008; Abreu et al., 2014) could indicate that DMH neurons under gabaergic influence are involved in the modulation of stress-evoked cardiovascular responses as a local facilitatory component in the neural circuitry. In the present study, the inactivation of the DMH with CoCl_2_ increased the baseline cardiovascular parameters at rest condition; however, the maximal RS-evoked BP an HR (raw values) increase was the same in vehicle-treated and cobalt-treated animals, and consequently, the change from baseline (ΔMAP and ΔHR) was smaller in the CoCl_2_-treated group when compared to the vehicle-treated one, a result that could suggest a DMH facilitatory influence on the cardiovascular response caused by RS. Alternatively, the attenuation of the RS-evoked cardiovascular response observed in this study could be explained by the baseline alteration caused by the CoCl_2_ microinjection into the DMH, and consequently the existence of a ceiling effect for RS-evoked cardiovascular responses. However, previous studies have demonstrated that the tachycardic (Tavares and Corrêa, 2006; Crestani Alves et al., 2009; Fortaleza et al., 2009) response could be still potentiated after the CoCl_2_ microinjection in different central nervous system areas, arguing the idea of the existence of a ceiling effect, and favoring the idea that DMH inhibition reduces the RS-evoked BP and HR responses. Nonetheless, further studies are necessary to clarify the issue.

Besides its involvement in cardiovascular modulation, the DMH is also recognized as a thermoregulatory structure. In this context, we found that inhibition of the DMH by local microinjection of CoCl_2,_ prevented the RS-evoked fall in tail temperature in rats, when compared to the vehicle-treated animals, suggesting that DMH plays a facilitatory role on the sympathetic control of tail artery territory, which is associated with the decrease of tail temperature in response to RS. The thermoregulatory role of the DMH can be justified by the neural input that DMH receives from the medial preoptic area (mPOA), a well-recognized thermoregulatory center (Thompson and Swanson, 1998; Hunt et al., 2010). Supporting the idea of a DMH modulation of the tail temperature during RS, our group has reported previously that microinjection of CoCl_2_ into the mPOA impaired the reduction in tail temperature caused by this stress model (Fassini et al., 2017), reinforcing the existence of a neuronal communication between these two important thermoregulatory centers, during the exposure to acute RS.

In relation to RS-evoked neuroendocrine responses, the increases in the plasma levels of corticosterone and oxytocin were not affected by the pretreatment of the DMH with CoCl_2_, when compared to the vehicle-stressed group. No changes in vasopressin plasma content were observed in either vehicle or CoCl_2_-treated animals, suggesting that DMH does not modulate the neuroendocrine response observed during the exposure to RS. Nonetheless, in a recent study, it has been reported that activation of the serotonergic neurotransmission in the DMH impaired the increase in costicosterone plasmatic levels caused by acute RS (Stamper et al., 2017), thus suggesting that selective neurotransmissions in the DMH can modulate the neuroendocrine response caused by RS.

In conclusion, the results from the inactivation of DMH synaptic function by local microinjections of CoCl_2_ suggests the involvement of the DMH in the maintenance of cardiovascular tonus, playing an inhibitory influence on the BP and HR baseline under rest conditions at home cage, and a facilitatory influence on the baseline vasopressin release into circulation, without affecting the baseline tail temperature, corticosterone and oxytocin plasma levels under rest conditions at the home-cage. In addition, results indicating that DMH that inactivation with CoCl_2_ interfered with RS-evoked BP and HR increase, and prevented the RS-evoked reduction in tail temperature without affecting the RS-evoked increase in plasma corticosterone and oxytocin plasma levels, suggest the involvement of the DMH in the neural circuitry controlling RS-evoked cardiovascular responses, and clearly indicate that functional integrity of this area is essential for the generation of the to the RS-evoked fall in tail temperature.

## Data Availability Statement

All datasets generated for this study are included in the article.

## Ethics Statement

The animal study was reviewed and approved by the University of São Paulo Ethical Committee - Animal Experimentation Control (CEUA) (no) 221/2018.

## Author Contributions

TB and FC conceived and designed this study. TB, SL-A, IB-F, and EF performed the experiments. TB analyzed the data. TB, JA-R, and FC interpreted the results of experiments. TB prepared the figures. TB drafted the manuscript. TB and FC edited and revised the manuscript. JA-R and FC approved the final version of the manuscript.

## Funding

This work was supported by grants from CNPq (474477/ 2013-4) and FAEPA.

## Conflict of Interest

The authors declare that the research was conducted in the absence of any commercial or financial relationships that could be construed as a potential conflict of interest.

## Abbreviations

DMH, Dorsomedial hypothalamus; PVN, paraventricular nucleus of the hypothalamus; mPOA, medial preoptic area; MAP, mean arterial pressure; BP, blood pressure; HR, heart rate; ACTH, adrenocorticotropic hormone.
